# Long-term outcomes of pulmonary atresia with ventricular septal defect by different initial rehabilitative surgical age

**DOI:** 10.3389/fcvm.2023.1189954

**Published:** 2023-10-16

**Authors:** Jianrui Ma, Tong Tan, Shuai Zhang, Wen Xie, Yinru He, Miao Tian, Zichao Tujia, Xinming Li, Xiaobing Liu, Jimei Chen, Jian Zhuang, Jianzheng Cen, Shusheng Wen, Haiyun Yuan

**Affiliations:** ^1^ Shantou University Medical College, Shantou, China; ^2^ Department of Cardiovascular Surgery, Guangdong Cardiovascular Institute, Guangdong Provincial People’s Hospital, Guangdong Academy of Medical Sciences, Southern Medical University, Guangzhou, China; ^3^ Guangdong Provincial Key Laboratory of South China Structural Heart Disease, Guangzhou, China; ^4^ Department of Cardiovascular Surgery Center, Beijing Anzhen Hospital, Capital Medical University, Beijing Institute of Heart, Lung and Blood Vascular Diseases, Beijing, China

**Keywords:** pulmonary atresia, age, rehabilitation, complete repair, timing

## Abstract

**Background:**

There is a lack of evidence guiding the surgical timing selection in pulmonary atresia with ventricular septal defect. This study aims to compare the long-term outcomes of different initial rehabilitative surgical ages in patients with pulmonary atresia with ventricular septal defect (PAVSD).

**Methods:**

From January 2011 to December 2020, a total of 101 PAVSD patients undergoing the initial rehabilitative surgery at our center were retrospectively reviewed. Receiver-operator characteristics curve analysis was used to identify the cutoff age of 6.4 months and therefore to classify the patients into two groups. Competing risk models were used to identify risk factors associated with complete repair. The probability of survival and complete repair were compared between the two groups using the Kaplan-Meier curve and cumulative incidence curve, respectively.

**Results:**

The median duration of follow-up was 72.76 months. There were similar ΔMcGoon ratio and ΔNakata index between the two groups. Multivariate analysis showed that age ≤6.4 months (hazard ratio (HR) = 2.728; 95% confidence interval (CI):1.122–6.637; *p* = 0.027) and right ventricle-to-pulmonary artery connection (HR = 4.196; 95% CI = 1.782–9.883; *p* = 0.001) were associated with increased probability of complete repair. The cumulative incidence curve showed that the estimated complete repair rates were 64% ± 8% after 3 years and 69% ± 8%% after 5 years in the younger group, significantly higher than 28% ± 6% after 3 years and 33% ± 6% after 5 years in the elder group (*p* < 0.001). There was no significant difference regarding the estimated survival rate between the two groups.

**Conclusion:**

Compared with those undergoing the initial rehabilitative surgery at the age >6.4 months, PAVSD patients at the age ≤6.4 months had an equal pulmonary vasculature development, a similar probability of survival but an improved probability of complete repair.

## 1. Introduction

Pulmonary atresia with ventricular septal defect (PAVSD) is a rare and complex form of congenital heart abnormality that is estimated to occur in 4.2–10 of 100,000 live births (1). The surgical management presents significant challenges due to erratic anatomic and morphologic characteristics. There are two approaches to achieving one-stage or multi-stage complete repair in terms of reconstruction of right ventricle-to-pulmonary artery continuity and VSD closure: the unifocalization strategy and the rehabilitation strategy. The unifocalization entails dissecting the major aortopulmonary collateral arteries (MAPCAs) and anastomosing them to each other and subsequently to the pulmonary artery, while rehabilitation aims to promote the development of pulmonary artery by systemic-to-pulmonary artery shunt or right ventricle-to-pulmonary artery (RV-PA) connection.

Previous studies have demonstrated that both of them would improve PAVSD patients' outcomes in recent decades (2, 3). However, these patients might show heterogeneous clinical outcomes and prognosis (4, 5), highlighting the necessity of appropriate selection of surgical indications and timing. The indication for surgery relies on the extent of pulmonary artery development (6), while the optimal timing for the initial surgery remains unclear. Some PAVSD patients present to the hospital and receive the initial surgical treatment late in our country because of an absence of significant cyanosis, limited specialty pediatrics, family's financial situation, and bed availability, which may adversely impact the long-term outcomes.

Given this, we conducted a retrospective review of PAVSD patients who underwent initial rehabilitative surgery at our center. The patients were classified into two groups based on an age cutoff of 6.4 months. We then compared the perioperative and long-term outcomes between these two groups.

## 2. Methods

### 2.1. Patients and design

**Figure 1:**
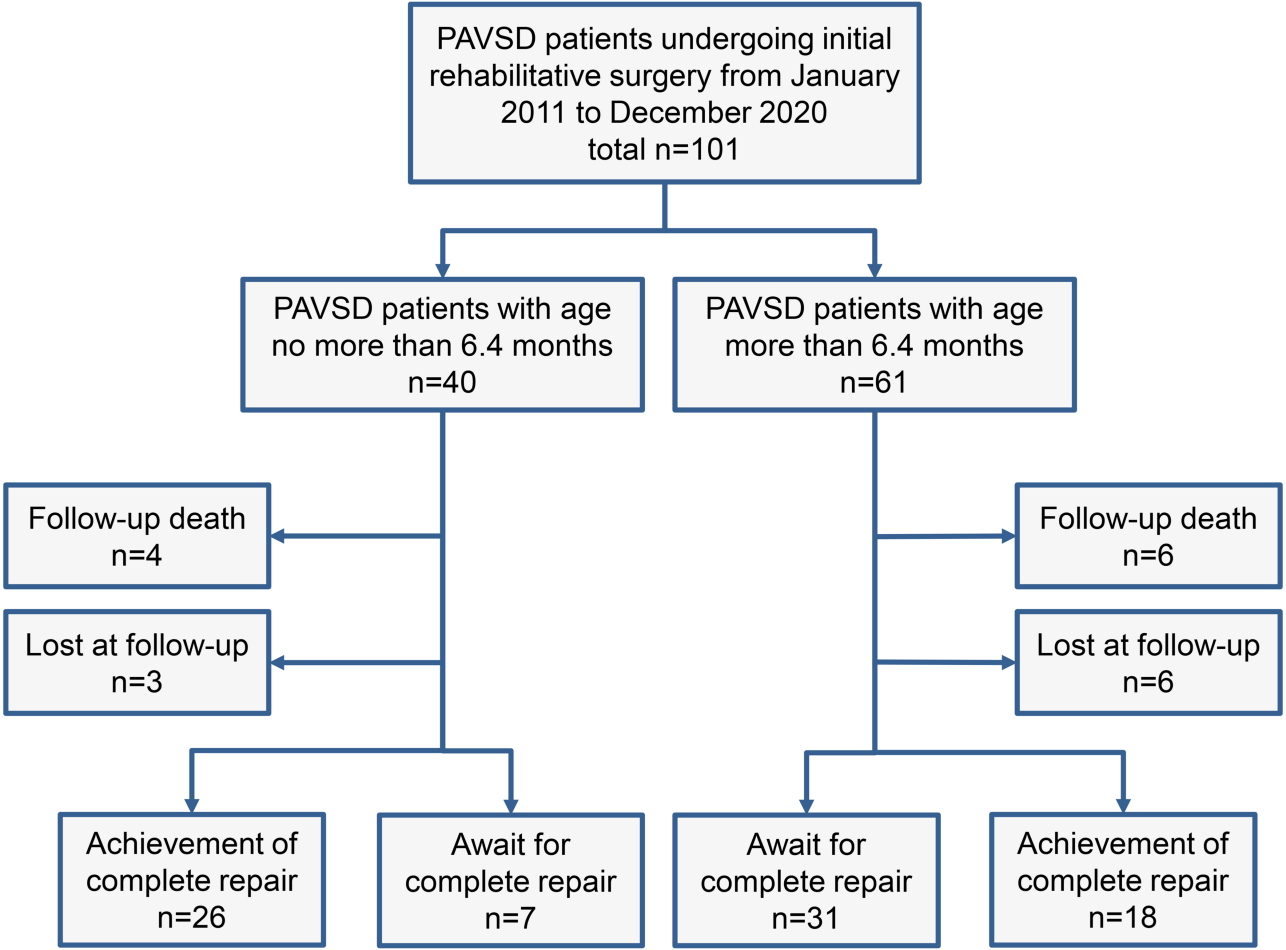
Flow diagram of follow-up and outcome of PAVSD patients undergoing initial rehabilitative surgery. PAVSD, pulmonary atresia with ventricular septal defect.

The present study was approved by the Guangdong Provincial People's Hospital ethics committee (No. GDREC2019338H(R2)) on September 17th, 2019. A total of 101 PAVSD patients undergoing the initial rehabilitative surgery, either through systemic-to-pulmonary artery shunt or right ventricle-to-pulmonary artery connection, between January 2011 to December 2020 at Guangdong Provincial People's Hospital were retrospectively reviewed and included in this study. PAVSD patients with concurrent complex intracardiac abnormalities or those who were not suitable for bi-ventricle repair were excluded from the study. Of 101 patients, those who were referred to our hospital early underwent the initial rehabilitative surgery in time after a full assessment of clinical conditions such as pulmonary artery development, MAPCAs distribution, and pulmonary flow. However, some were delayed in undergoing the initial rehabilitative surgery, predominantly due to the late referral to our hospital. As shown in Figure 1, all patients were divided into 2 groups according to age. 40 PAVSD patients underwent the initial rehabilitative surgery at the age ≤6.4 months, while 61 patients underwent it at the age >6.4 months. The difference in initial rehabilitative age would thereby result in a significant difference in weight and body surface areas (BSA) between the two groups. Additionally, patients in the elder group were referred to our hospital late possibly on account of inapparent symptoms of hypoxemia. MAPCAs that can provide sufficient pulmonary flow and alleviate symptoms of cyanosis were thereby more frequently observed in the elder group (Table 1).

**Table 1:** Baseline characteristics and clinical data.

Variables	Total *n* = 101	Age ≤6.4 months *n* = 40	Age >6.4 months *n* = 61	*p*
Age (month), median (IQR)	9.83 (2.94–45.19)	1.90 (0.73–3.65)	33.37 (12.84–80.04)	<0.001
Male	56 (55.4%)	27 (67.5%)	29 (47.5%)	0.048
Weight (kg), median (IQR)	7.5 (4.5–12.3)	3.8 (2.7–5.2)	11.0 (8.0–17.0)	<0.001
BSA (m^2^), median (IQR)	0.37 (0.24–0.57)	0.22 (0.18–0.26)	0.52 (0.39–0.74)	<0.001
Types				0.092
A	44 (43.6%)	22 (55.0%)	22 (36.1%)	
B	50 (49.5%)	17 (42.5%)	33 (54.1%)	
C	7 (6.9%)	1 (2.5%)	6 (9.8%)	
PaO_2_, median (IQR)	73.0 (66.0–80.0)	70.0 (60.0–84.5)	73.0 (70.0–78.5)	0.362
CT imaging				
Presence of central pulmonary artery	94 (93.1%)	39 (97.5%)	55 (90.2%)	0.308
Presence of MAPCAs	41 (40.6%)	6 (15%)	35 (57.4%)	<0.001
McGoon ratio, mean ± SD	0.88 ± 0.36	1.00 ± 0.21	0.80 ± 0.42	0.003
Nakata index, median (IQR)	59.39 (35.69–92.20)	77.87 (51.38–96.82)	54.86 (27.45–83.90)	0.023
Rehabilitative approach				0.802
Pulmonary-to-pulmonary artery shunt	54 (53.5%)	22 (55%)	32 (52.5%)	
RV-PA connection	47 (46.5%)	18 (45%)	29 (47.5%)	
Concurrent unifocalization	10 (9.9%)	1 (2.5%)	9 (14.8%)	0.094

BSA, body surface area; CT, computed tomography; MAPCAs, major aortopulmonary collateral arteries; SD, standard deviation; RV-PA connection, right ventricle to pulmonary artery connection.

The medical history, imaging examination, surgery information, and perioperative records were all extracted and collected. The McGoon ratio and Nakata index were calculated on CT imaging to assess the pulmonary vessel growth, as described by Jia et al. (7). The Nakata index and the McGoon ratio were calculated under CT imaging through the following formulas: the sum of the bilateral pulmonary artery cross-section areas divided by the BSA and the sum of bilateral pulmonary artery diameter divided by the descending aorta diameter at the level of the diaphragm, respectively. Therefore, the Nakata index and McGoon ratio were lower in the elder group possibly because the growth of the pulmonary artery was significantly slower than the growth of BSA and descending aorta in this context as the age increased (Table 1).

### 2.2. Operation strategies and techniques

The technique for establishing RV-PA connection was previously described in detail (8). Briefly, the median sternotomy was performed followed by the initiation of cardiopulmonary bypass. When the main pulmonary artery was presented, a transannular patch would be used to suture to the edges of the pulmonary artery longitudinal incision and RV incision. In cases where the pulmonary artery was not present, alternative methods such as using the autologous pericardial roll, Gore-tex conduit, bovine pericardial roll, or jugular vein conduit were employed to reconstruct the RV-PA connection. The diameter and size of the RV-PA connection were approximately 5–12 mm, primarily determined by the oxygen saturation variation and patients' weight.

In contrast, the establishment of a systemic-to-pulmonary shunt could be performed without the use of a cardiopulmonary bypass, including a central shunt and a modified Blalock-Taussig (B-T) shunt. The technique for the central shunt was described by Gates et al. in 1998 (9). It involved making a longitudinal incision in the pulmonary artery branch after applying a side-biting clamp, which was then anastomosed to a Gore-Tex conduit. The ascending aorta was also clamped by a side-biting clamp to create a proximal orifice with an appropriate diameter. The final proximal anastomosis was finished in either a side-to-end or side-to-side manner. The technique to create a modified B-T shunt was reported by Leval et al. (10). Briefly, a Gore-Tex conduit was adopted to anastomose the innominate artery or subclavian artery and the main pulmonary artery.

When satisfactory pulmonary artery growth was obtained, the complete repair would be performed by reconstruction of the right ventricle outflow tract using a valved conduit or patch as well as VSD closure.

### 2.3. Follow-up and definition

All patients were scheduled for regular follow-up visits at the outpatient clinic at 1, 3, 6, and 12 months after the rehabilitative surgery, followed by annual visits. CT would be performed if there was satisfactory pulmonary vessel growth or suspicious vessel stenosis. A recent phone contact has also been performed to investigate their latest clinical status including NYHA heart function class, complication, survival, complete repair, and so on. The date of the recent phone contact was November 29th, 2022. Those who never came to visit the outpatient clinic in the entire postoperative period or only came to visit the outpatient clinic for few times in the initial postoperative period and could not be contacted by the recent phone call were considered to be lost to follow-up. The endpoints were defined as death or complete repair during the follow-up. In-hospital mortality or follow-up mortality were defined as death before discharge and during the follow-up, respectively. The ΔMcGoon ratio/ΔNakata index was defined as the difference between the McGoon ratio/Nakata index before the complete repair and before the initial rehabilitative surgery.

### 2.4. Statistical analysis

**Figure 2:**
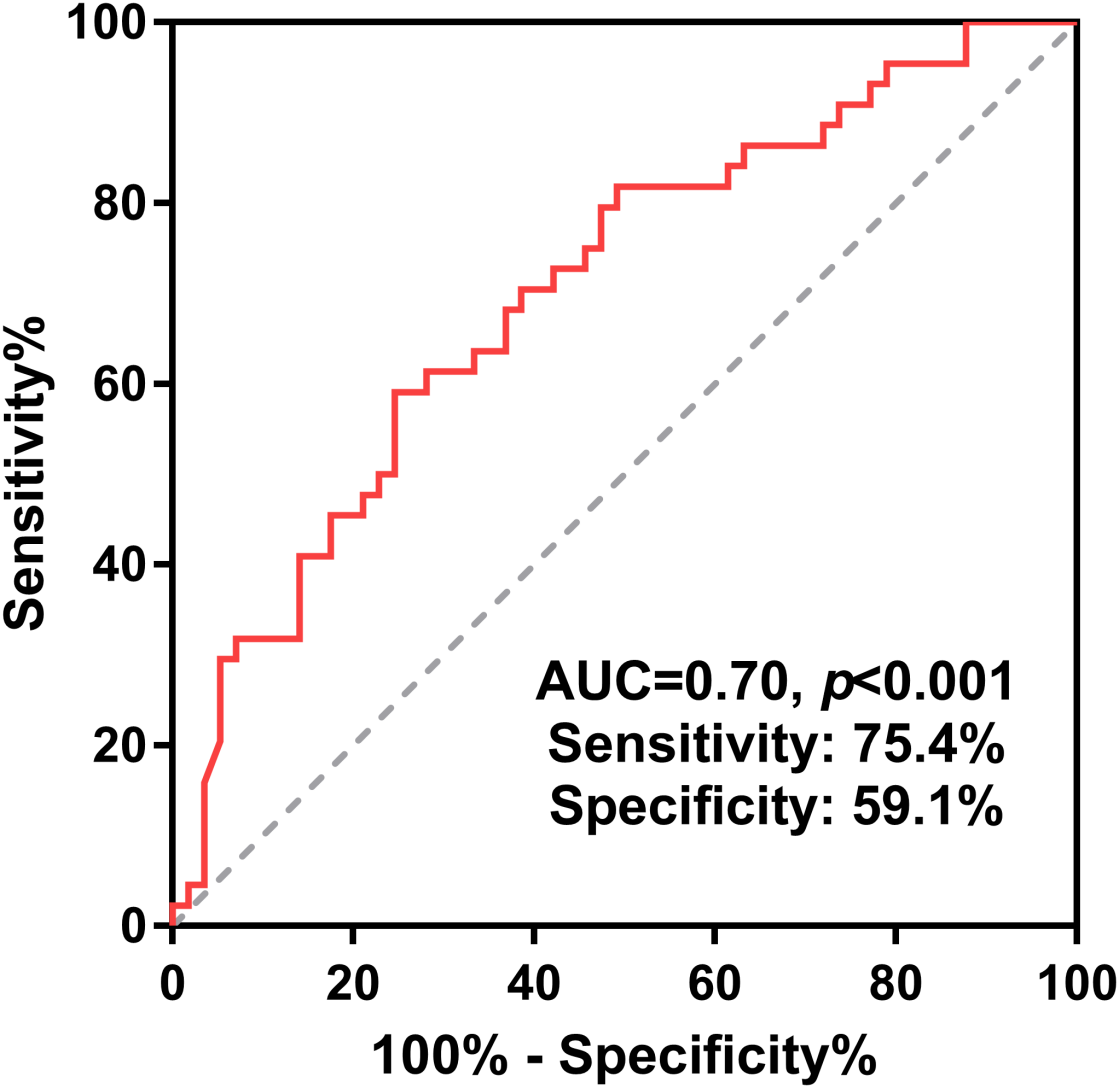
Receiver-operating characteristic curve for complete repair by age was adopted to determine the cut-off age and thereby divide all patients into two groups. PAVSD, pulmonary atresia with ventricular septal defect.

Continuous variables were presented as means with standard deviations if normally distributed or medians with ranges if abnormally distributed. Categorical variables were presented as numbers with percentages. The receiver-operator characteristics (ROC) curve was created with the area under the curve (AUC) to determine the cut-off age. As shown in Figure 2, the cutoff age for complete repair was 6.4 months (AUC = 0.70, *p* < 0.001), which was therefore used to classify all the PAVSD patients into two groups (age ≤6.4 months vs. age >6.4 months). The student's *t*-test or Mann–Whitney *U*-test was applied to compare the continuous variables with normal distribution or abnormal distribution between the two groups. The chi-squared test was used to compare the categorical variables between the two groups. Competing risk regression analysis were performed for the complete repair (with death as a competing event). The postoperative survival was estimated by the Kaplan–Meier curve with the log-rank test. The complete repair was estimated by cumulative incidence curve with Gray's test. All statistical analysis was conducted using SPSS version 24.0. A *p*-value less than 0.05 was considered to be significant.

## 3. Results

### 3.1. Baseline clinical characteristics

The baseline clinical characteristics were summarized in the Table 1. The entire cohort had a median age of 9.83 months. Of 101 PAVSD patients, 56 (55.4%) were male. In the younger group, 67.5% were male, while in the elder group, 47.5% were male. The median age of the younger group and elder group were 1.90 and 33.37 months, respectively. No significant difference was observed between the two groups in terms of types, PaO2, presence of central pulmonary artery, rehabilitative approach, and concurrent unifocalization (*p* > 0.05). Compared with the elder group, the younger group showed significantly lower weight, BSA, presence of MAPCAs, McGoon ratio, and Nakata index (*p* < 0.05).

### 3.2. Perioperative and postoperative outcome

Two groups showed a similar time of cardiopulmonary bypass and aorta cross-clamping, as shown in Table 2. There was no in-hospital mortality in both two groups. In contrast to the elder group, the younger group was associated with a significantly higher incidence of delayed sternal closure (*p* = 0.020) and longer length of hospital stay (*p* = 0.016). The mean follow-up time of the entire cohort was 72.76 months. The follow-up mortality was similar, with 4 and 6 deaths occurring in the younger group and the elder group, respectively. There was also no significant difference in the ΔMcGoon ratio and ΔNakata index between the two groups. Of note, inter-stage reintervention including percutaneous balloon pulmonary valvuloplasty, percutaneous pulmonary artery stent implantation, and shunt/conduit replacement were similar between the two groups (Table 2). The pulmonary artery growth in PAVSD patients with or without a central pulmonary artery was also shown in Supplementary Table S1.

**Table 2:** Initial perioperative data and postoperative follow-up.

Variables	Total *n* = 101	Age ≤6.4 months *n* = 40	Age >6.4 months *n* = 61	*p*
Perioperative				
CPBT (min), median (IQR)	46.00 (0.00–109.50)	45.50 (0.00–95.75)	78.00 (0.00–123.00)	0.442
ACCT (min), median (IQR)	0.00 (0.00–62.00)	0.00 (0.00–58.50)	0.00 (0.00–62.00)	0.679
Delayed sternal closure	17 (16.8%)	11 (27.5%)	6 (9.8%)	0.020
In-hospital mortality	0	0	0	–
Length of hospital stay (d), median (IQR)	29.0 (22.0–42.0)	34.0 (27.0–48.0)	28.0 (21.0–37.5)	0.016
Postoperative				
Follow-up period (month), median (IQR)	72.76 ± 36.03	75.38 ± 40.53	71.05 ± 32.98	0.558
Inter-stage reintervention				
Percutaneous balloon pulmonary valvuloplasty	2 (2.0%)	1 (2.5%)	1 (1.6%)	>0.999
Percutaneous pulmonary artery stent implantation	3 (3.0%)	0	3 (4.9%)	0.410
Shunt/conduit replacement	20 (19.8%)	8 (20.0%)	12 (19.7%)	0.968
Follow-up mortality	10 (9.9%)	4 (10%)	6 (9.8%)	0.978
McGoon ratio before complete repair, mean ± SD	1.65 ± 0.48	1.67 ± 0.47	1.61 ± 0.50	0.679
ΔMcGoon ratio, mean ± SD	0.72 ± 0.46	0.64 ± 0.49	0.84 ± 0.41	0.160
Nakata index before complete repair, median (IQR)	200.92 (108.36–319.80)	207.81 (98.54–354.80)	200.92 (120.97–249.22)	0.634
ΔNakata index, median (IQR)	92.19 (50.78–181.06)	118.65 (52.48–278.62)	85.91 (48.23–139.59)	0.189
Complete repair	44	26	18	–

CPBT, cardiopulmonary bypass time; ACCT, aorta cross-clamping time.

### 3.3. Survival analysis for complete repair and survival

**Figure 3:**
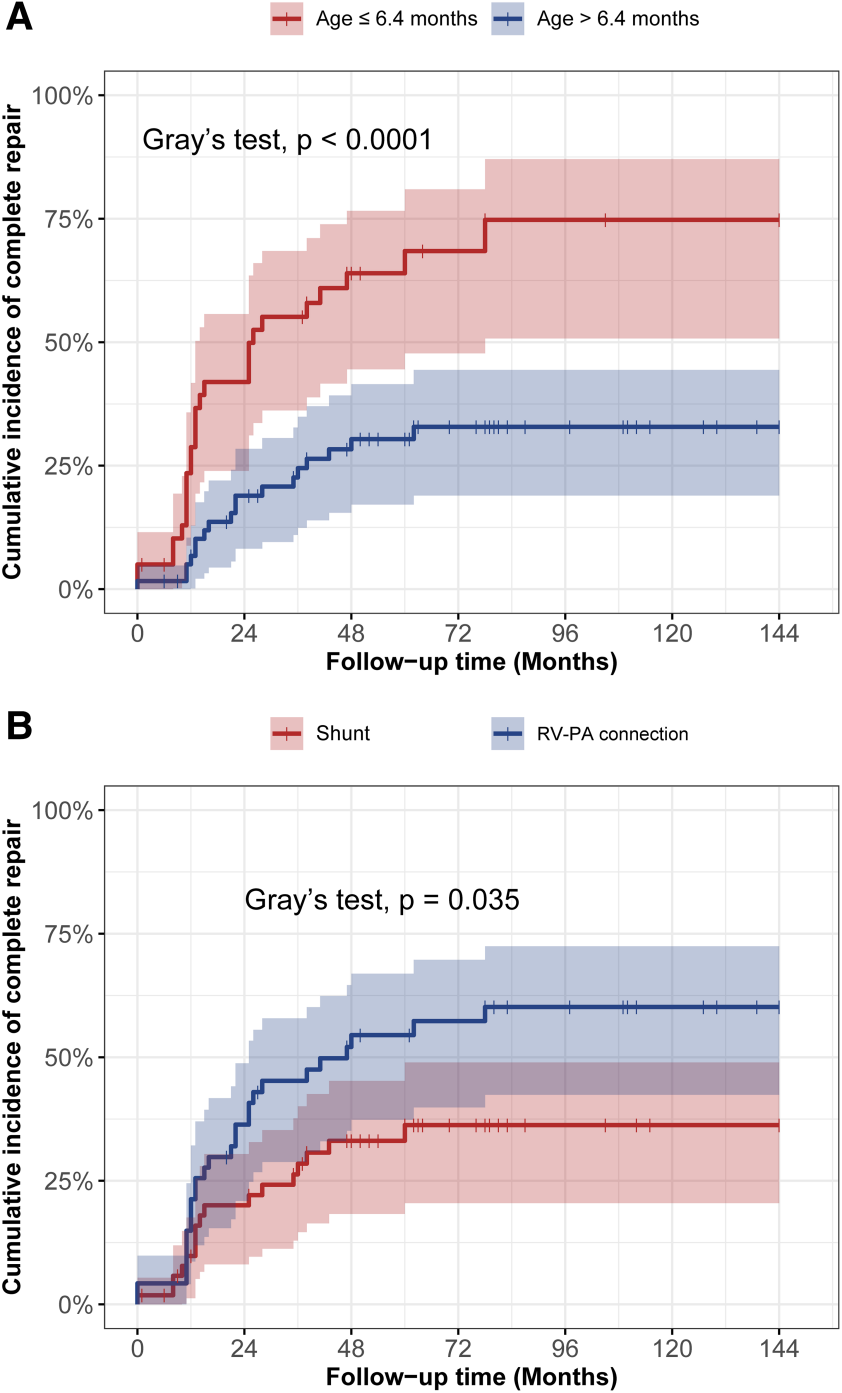
The cumulative incidence curve analysis of complete repair between different age groups (**A**) and different rehabilitation strategies (**B**).

**Figure 4:**
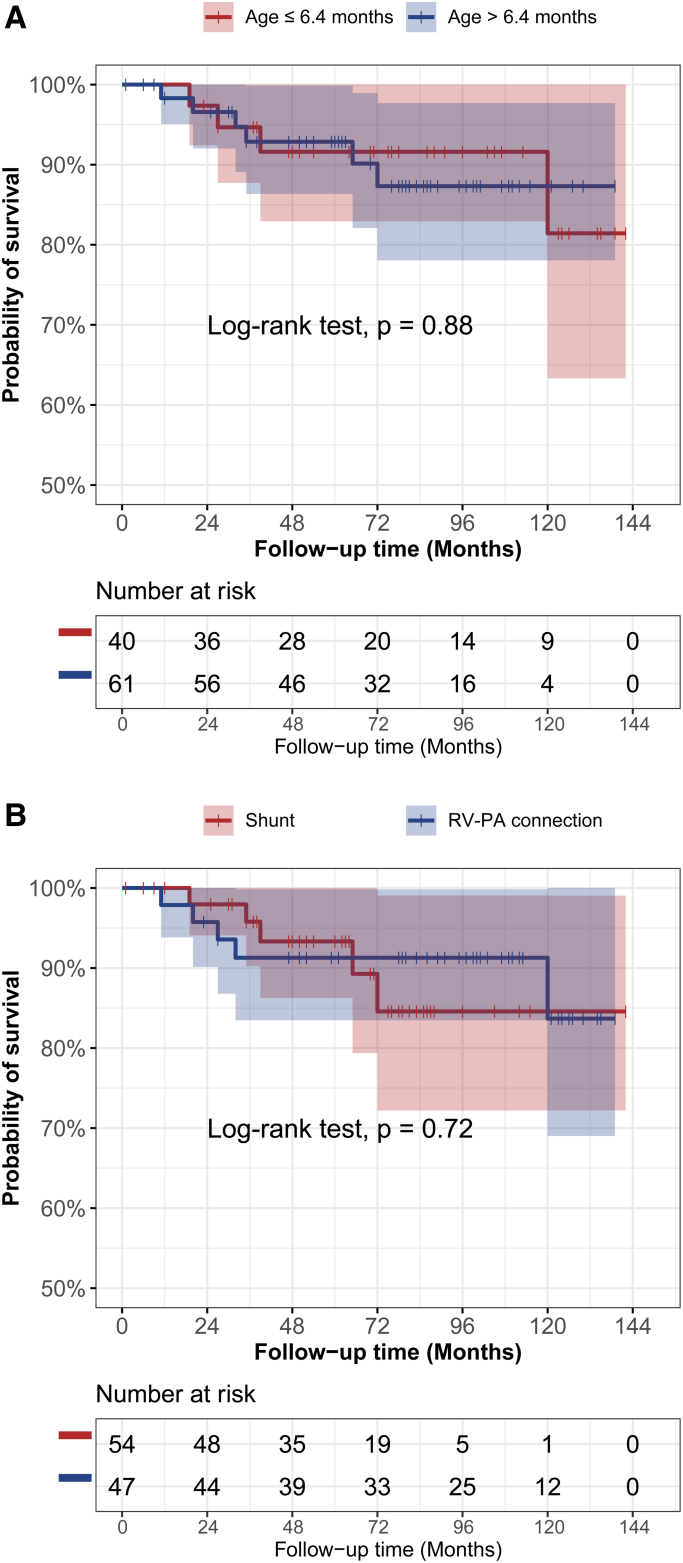
The kaplan-meier curve analysis of survival between different age groups (**A**) and different rehabilitative surgery groups (**B**).

In the Figure 3, the cumulative incidence curve revealed that patients in the younger group had a significantly higher probability of complete repair than those in the elder group (*p* < 0.001). The overall 3-year and 5-year complete repair rates in the younger group were 64% ± 8% and 69% ± 8%, respectively, in contrast to 28% ± 6% and 33% ± 6% in the elder group, respectively. Additionally, Patients initially undergoing RV-PA connection also had a significantly higher probability of complete repair in contrast to those undergoing systemic-to-pulmonary artery shunt (*p* = 0.035). No significant differences were observed in terms of the probability of survival by age groups (Figure 4A) or rehabilitative surgery groups (Figure 4B).

### 3.4. Multivariate analysis for complete repair

The univariate regression analysis showed that age ≤6.4 months, weight, presence of MAPCAs, Nakata index, and RV-PA connection were predictors of complete repair (Table 3). These predictors and clinically significant factors in terms of PaO2 and McGoon ratio were included to perform the multivariate analysis. Age ≤6.4 months (hazard ratio (HR) = 2.728; 95% confidence interval (CI):1.122–6.637; *p* = 0.027), McGoon ratio (HR = 0.105; 95% CI = 0.025–0.439; *p* = 0.002), Nakata index (HR = 1.016; 95% CI = 1.005–1.027; *p* = 0.005), and RV-PA connection (HR = 4.196; 95% CI = 1.782–9.883; *p* = 0.001) were identified as independent factors associated with complete repair.

**Table 3:** Univariate and multivariate analysis for complete repair.

Variables	Univariate analysis			Multivariate analysis		
	HR	95% CI	*p*	HR	95% CI	*p*
Age ≤6.4 months	3.158	1.724–5.786	0.000	2.728	1.122–6.637	0.027
Male	1.383	0.753–2.537	0.296			
Weight, kg	0.902	0.842–0.967	0.003	0.927	0.852–1.009	0.080
PaO_2_	0.980	0.958–1.003	0.091	0.988	0.966–1.010	0.289
Presence of MAPCAs	0.470	0.242–0.913	0.026	1.298	0.578–2.914	0.528
McGoon ratio	1.455	0.673–3.146	0.341	0.105	0.025–0.439	0.002
Nakata index	1.004	0.997–1.011	0.287	1.016	1.005–1.027	0.005
RV-PA connection	1.892	1.030–3.474	0.040	4.196	1.782–9.883	0.001

MAPCAs, major aortopulmonary collateral arteries; RV-PA connection, right ventricle to pulmonary artery connection.

## 4. Discussion

The ideal treatment and management for PAVSD patients, particularly regarding the determination of surgical timing, remains undefined. To the best of our knowledge, this is the first study to identify the cutoff age at initial surgery for PAVSD to evaluate its impact on clinical outcomes. In this study, our findings showed that PAVSD patients in the younger group had a significantly improved complete repair possibility than those in the elder group. What's more, despite a higher incidence of delayed sternal closure, the younger group had similar and comparable pulmonary artery growth and mortality compared to the elder group.

In our study, the estimated complete repair rate in the younger group (median age: 1.90 months) was 64% ± 8% after 3 years and 69% ± 8% after 5 years, which was significantly higher than the 28% ± 6% after 3 years and 33% ± 6% after 5 years in the elder group (median age: 33.37 months). In general, our estimated complete repair rates were somewhat lower than those in the previous studies (1, 11, 12). We attributed it to the criteria differences in patient enrollment. Previous studies regarding the complete repair rate in such individuals also showed great heterogeneity, probably due to the difference in initial operation age. In 2013, Zhang et al. retrospectively reviewed a total of 37 PAVSD patients with a mean age of 1.9 years old to investigate the outcome of RV-PA connection as an initial rehabilitative surgery. Seventeen patients (45.9%) eventually achieved complete repair (13). They also performed another study among 69 patients with pulmonary atresia, ventricular septal defect, and hypoplastic pulmonary artery with a mean age of 1.8 years old in 2016 to determine the effect of a multi-stage pulmonary artery rehabilitation strategy (3). The overall 3-year complete repair rate was estimated to be 60.1%. In contrast, a higher complete repair rate would be achieved in those younger than the aforementioned cohort (14). Gerelli et al. investigated the RV-PA connection in 107 PAVSD patients with a median age of 10 days, showing that 47 patients (81%) underwent a complete repair (8). Choi et al. also performed RV-PA connection to rehabilitate the pulmonary artery in 13 patients at the mean age of 17.9 ± 15.3 days. All patients that survived the RV-PA connection eventually achieved complete repair after a mean interval of 13.1 months (15). Therefore, the age at initial surgery had a crucial impact on the outcome of PAVSD patients indeed. This might be explained by several reasons as follows. First, the PAVSD patients in the younger group themselves possess more potency in pulmonary vasculature development after receiving rehabilitative surgery than those in the elder group, resulting in more probability of eventual complete repair. Some authors also considered that an age of less than half one year was a more appropriate period for the development of pulmonary arteries (13). Secondly, different initial pulmonary artery statuses probably in part contribute to the different results. The McGoon ratio and Nakata index in the younger group were significantly higher than those in the elder group, indicating better pulmonary artery development. It has been demonstrated that a higher McGoon ratio was associated with an increased probability of complete repair (16).

There were no deaths in hospital. The follow-up mortality was also comparable between the younger group (10%) and the elder group (9.8%), which was consistent with the previous investigation (17–20). Macalister et al. reported a mortality rate ranging from approximately 6%–20% during a median follow-up period of 10.5 years (21). In the younger group, a higher incidence of delayed sternal closure and longer lengths of hospital stay were observed. We attributed these to the cardiovascular structural and functional vulnerability as well as relative technical difficulty. Although possibly compromising the postoperative in-hospital performance, early rehabilitative surgery would not increase the risk of mortality.

For Tetralogy of Fallot selecting for complete repair, it is recommended to perform surgical correction between the age of 3 months and 6 months with a Class IIa recommendation (22). Though PAVSD is considered an extreme type of tetralogy of Fallot, there was a lack of consensus to guide the selection of surgical indication and timing. In our study, we found the age at initial rehabilitative surgery was a key predictor for a final complete repair. Using ROC analysis, we identified 6.4 months as the cutoff age, showing the PAVSD patients early received the rehabilitative surgery had a better prognosis in terms of comparable probability of survival but improved probability of complete repair. Hence, if the clinical status and surgical technique are feasible, PAVSD patients in an attempt to initially adopt the rehabilitative strategy are recommended to receive the first stage surgery before approximately six months old. In this context, however, a concern was raised that the initial rehabilitative surgery performed in the younger patients would potentially lead to an increased risk of replacement of a bigger shunt/conduit to meet the demand as patients grew older. Our preliminary analysis showed that the incidence of shunt/conduit replacement after the initial rehabilitative surgery was similar between the younger and elder groups. We assumed that the pulmonary artery would grow fast enough to achieve complete repair before the next-time replacement of a bigger shunt/conduit in PAVSD patients undergoing the initial rehabilitative surgery at the age ≤6.4 months.

In the multivariate analysis for complete repair, RV-PA connection was a positive predictor (HR = 4.196; 95% CI = 1.782–9.883; *p* = 0.001). Which kind of rehabilitative approach for PAVSD is more ideal remains highly debated. Fan et al. compared the perioperative and postoperative outcomes between 44 patients with systemic-to-pulmonary artery shunts and 54 patients with RV-PA connections. They demonstrated that systemic-to-pulmonary artery shunt resulted in better early postoperative outcomes in terms of shorter intubation time, length of intensive care unit stay, and length of hospital stay (23). Zhao et al. reviewed their institution's experience of 157 PAVSD patients with MAPCAs undergoing these two rehabilitative approaches. They found that RV-PA connection in contrast to systemic-to-pulmonary artery shunt was more advantageous in promoting pulmonary artery growth and final complete repair (24), which was in agreement with our findings. The benefits of RV-PA connection may be attributed, at least in part, to the adequate anterograde blood flow as well as the low risk associated with thrombosis or obstruction.

There were several limitations in our study. First, the results should be cautiously interpreted due to the retrospective one-single-center design. Secondly, the initial pulmonary vasculature status was different between the two groups, which may overestimate the impact of early surgery on outcomes. In addition, a more comprehensive and targeted analysis for comparing reintervention between the two groups should be performed to address safety concerns in younger patients. Finally, our variable reflecting the cardiac function performance improvement was limited. New York Heart Association Classification, clinical status, and oxygen saturation will be investigated in the future.

## 5. Conclusion

Despite similar pulmonary artery growth, PAVSD patients undergoing the initial rehabilitative surgery at the age ≤6.4 months had an improved possibility of complete repair without increasing risk of mortality and reintervention in contrast to those at the age >6.4 months. These findings aid in surgical timing selection, benefiting the evidence supplement of the management algorithm in treating PAVSD patients.

## Data Availability

The raw data supporting the conclusions of this article will be made available by the authors, without undue reservation.
