# Supplementary material for: Long-term outcomes of pulmonary atresia with ventricular septal defect by different initial rehabilitative surgical age
Source: Front Cardiovasc Med. 2023 Oct 16;10:1189954. doi: 10.3389/fcvm.2023.1189954 (PMC10619854; doi:10.3389/fcvm.2023.1189954)
Supplement: Supplementary file 1 [file Table1.doc]

**Supplemental Table 1 Pulmonary artery growth in PAVSD patients with or without central Pulmonary artery**

| Variables | Total  n=101 | With central pulmonary artery  n=94 | Without central pulmonary artery  n=7 | *p* |
| --- | --- | --- | --- | --- |
| Follow-up period (month), median (IQR) | 72.76±36.03 | 87.05±35.38 | 45.50±19.09 | 0.422 |
| McGoon ratio before complete repair, mean±SD | 1.65±0.48 | 1.67±0.48 | 1.15±0.25 | 0.136 |
| ΔMcGoon ratio, mean±SD | 0.72±0.46 | 0.71±0.47 | 0.88±0.13 | 0.623 |
| Nakata index before complete repair, median (IQR) | 200.92 (108.36–319.80) | 215.38 (126.11–319.80) | 102.94 (99.49–106.39) | 0.164 |
| ΔNakata index, median (IQR) | 92.19 (50.78–181.06) | 105.65 (29.08–232.74) | 74.57 (42.74–106.39) | 0.672 |

PAVSD, pulmonary atresia with ventricular septal defect.
